# Conservative management of Type B aortic intramural hematoma guided by imaging surveillance

**DOI:** 10.1016/j.radcr.2026.01.081

**Published:** 2026-02-25

**Authors:** Farook AlBayati, Mohamed Hajalamin, Marienys Lara González, Yasmine El Hennawi, Mohammed Elsheikh

**Affiliations:** a Cavan General Hospital, 2 R212, H12 YP92, Cavan, Ireland; b University of Galway, University Road, H91TK33, Galway, Ireland; c St James’s Hospital, James Street, D08 NHY1, Dublin, Ireland; d Medical University of Sofia,15 Akademik Ivan Geshov Blvd, 1431, Sofia, Bulgaria; e University Hospital Galway, Newcastle Road, H91 YR71, Galway, Ireland

**Keywords:** Aortic intramural hematoma, Acute aortic syndrome, CT angiography, Chest imaging, TBIMH, IMH

## Abstract

Aortic intramural hematoma is a life-threatening condition within the spectrum of acute aortic syndromes and is often misdiagnosed. We present the case of a 76-year-old man who attended the Emergency Department with mild, atypical chest pain. Initial assessment and laboratory tests were reassuring, and a musculoskeletal cause was considered; however, high blood pressure and diagnostic uncertainty prompted computed tomography angiography. Imaging revealed type B aortic intramural hematoma. The patient was managed conservatively with strict control of blood pressure and heart rate and close imaging surveillance. Short-interval repeat computed tomography angiography demonstrated stability, supporting continued medical therapy. Over 3 years, the outcome was favorable with progressive resolution of the hematoma. This case highlights the importance of considering acute aortic pathology in older hypertensive patients with atypical pain, and it illustrates how imaging surveillance can guide safe conservative management.

## Background

Aortic intramural hematoma (IMH) is a highly morbid condition and part of the spectrum of acute aortic syndromes (AAS), alongside aortic dissection and penetrating aortic ulcer [1]. It is classified as Stanford type A or B based on the aortic segment involved [2]. IMH is characterized by hemorrhage within the media of the aortic wall, with or without intimal disruption. Type A IMH involves the ascending aorta and/or aortic arch, whereas type B IMH (TBIMH) originates distal to the left subclavian artery and involves the descending thoracic aorta [[3], [4], [5]].

IMH may be diagnostically challenging and can progress to aortic dissection or other complications; therefore, accurate diagnosis and risk stratification are essential to guide timely management [6]. Imaging has a central role in confirming the diagnosis, excluding dissection, and supporting management decisions in stable patients.

We present a case of TBIMH with extended imaging follow-up, highlighting the diagnostic pathway and the radiographic evaluation that supported conservative management.

## Case report

A 76-year-old man presented to the Emergency Department with pain “at the back of the chest” that began after intense gardening. The pain was mild, dull, and pleuritic, radiating to the left arm, worsening with shoulder movement, and relieved by rest. There were no associated shortness of breath, nausea, sweating, or dizziness. The patient reported intermittent recurrences of similar pain, which he had attributed to gardening activity. His medical history included hypertension and diabetes mellitus type 2, and dyslipidaemia for which he was taking simvastatin. He does not smoke and has no relevant family history. He was not on antihypertensives or diabetes medications.

On examination, the patient was alert, oriented, and appeared comfortable. He had a respiratory rate of 14 breaths per minute, a heart rate of 73 beats per minute with a regular rhythm, a temperature of 36.9°C, and maintained oxygen saturation on room air. Blood pressure was elevated at 195/100 mmHg, and there was no difference between the right and left arm readings. Cardiovascular and respiratory auscultations were unremarkable.

An electrocardiogram demonstrated a normal sinus rhythm without ischemic changes. Laboratory investigations, including full blood count, C-reactive protein, troponin, and D-dimer, were within normal limits. His symptoms improved following intravenous analgesia. Given the musculoskeletal nature of the pain, this was initially suspected as the cause. However, pulmonary embolism, coronary artery disease, and acute aortic syndrome (AAS) remained in the differential diagnosis. Although the pain was not typical for AAS, the patient’s hypertension and exertional onset warranted further investigation.

**Fig. 1:**
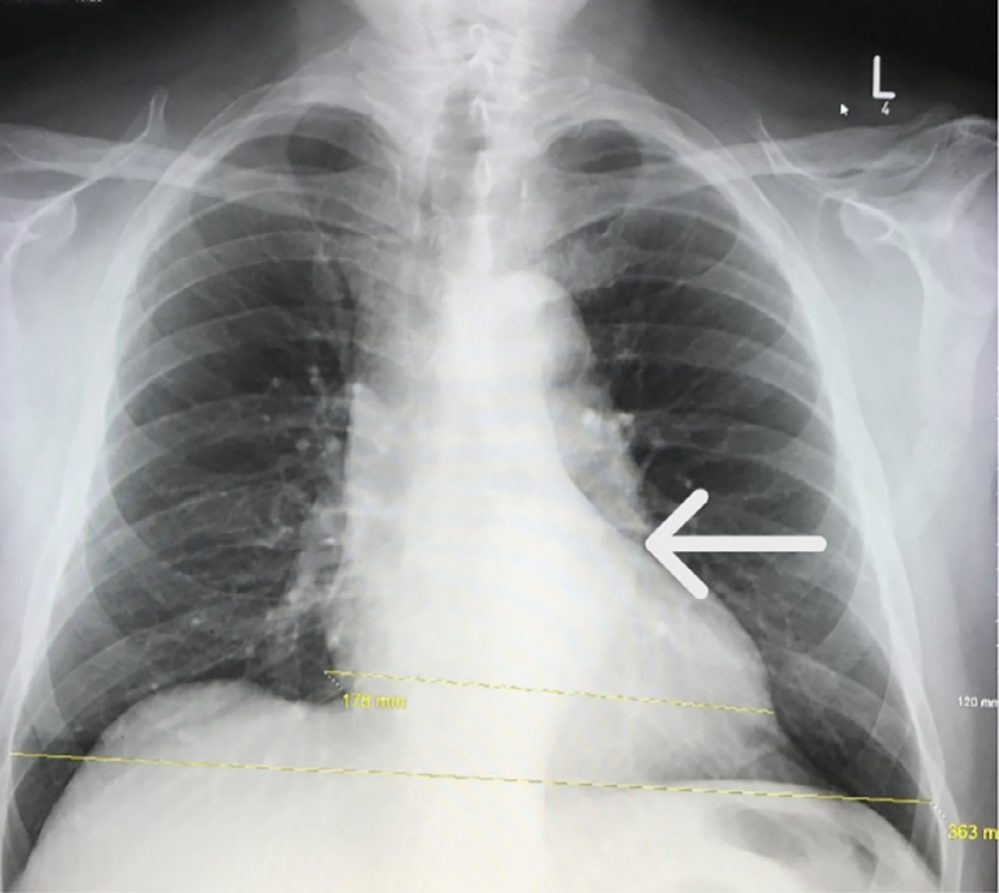
Posteroanterior chest X-ray showing normal cardiac size and mild unfolding of the aortic arch and tortuosity of the descending thoracic aorta (white arrow).

**Fig. 2:**
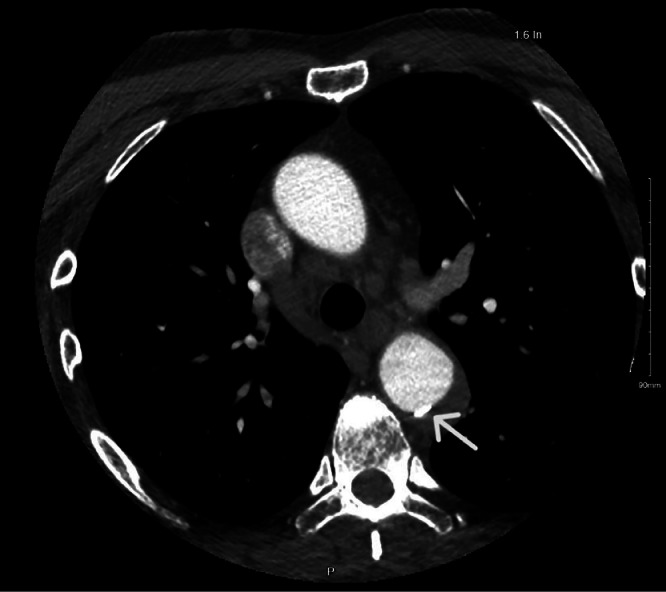
CT thoracic aortogram on day 1: axial cut at the level of the aortic arch showing ascending aorta ectasia (44mm). Normal aortic arch, and 41 mm descending aorta with fusiform posterior intraluminal thrombus containing an area of high density representing the acute intramural hematoma (white arrow). No dissection was identified.

Chest X-ray (Fig. 1) revealed a mildly unfolded aortic arch and tortuosity of the descending thoracic aorta. The patient was admitted and underwent a CT neck angiogram (Fig. 2), which showed ectasia of the ascending aorta (44 mm), a normal arch, and a 41 mm descending aorta with a fusiform posterior intraluminal thrombus containing a hyperdense area suspicious for IMH. No dissection was identified.

Following a multidisciplinary discussion with the vascular team, conservative management was initiated with strict control of blood pressure and heart rate. The patient was started on daily doses of ramipril 2.5 mg (later increased to 3.75 mg), lercanidipine 10 mg, and bisoprolol 1.25 mg to maintain systolic blood pressure below 120 mmHg and heart rate under 75 bpm. Additionally, metformin was commenced at 500 mg twice daily, and simvastatin was increased to 40 mg daily.

**Fig. 3:**
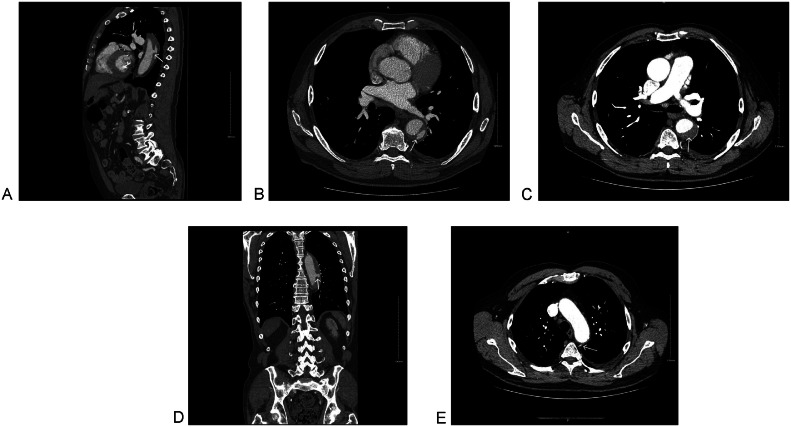
CT Angiogram of whole aorta on day 3 showing stable size of TBIMH with recanalization and connection with a left intercostal artery (white arrow). (A) Sagittal, para midline. (B) Axial at the level of the descending aorta. (C) Axial at the level of the great vessels. (D) Coronal at the level of the posterior mediastinum and (E) Axial at the level of the aortic arch.

A CT angiogram of the whole aorta repeated after 48 hours showed stable IMH size but new increased flow within the mural hematoma, with direct communication between the intramural blood and the aortic lumen at its cranial end, as well as a connection with a left intercostal vessel (Fig. 3). A repeat whole-aorta CT angiogram after 5 days showed no further progression. The patient was discharged with safety-netting advice and scheduled for outpatient follow-up in 3 months.

## Follow-up and outcomes

**Fig. 4:**
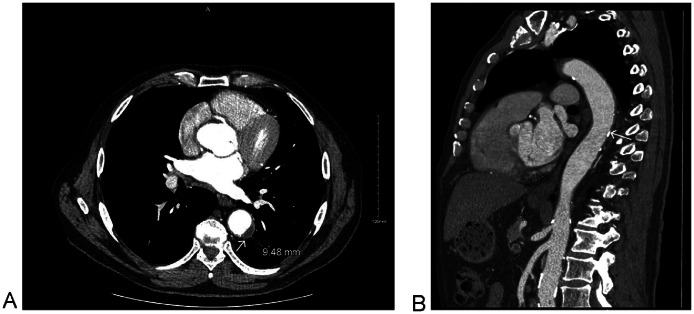
CT Angiogram of Whole Aorta after 3 months showing reduction in the size of TBIMH to 13 mm and reduction in maximum thickness of the thrombus to 9.5 mm (white arrow); where previously 30 mm and 14 mm, respectively. (A) Axial at the level of the descending aorta and (B) Sagittal para Medline.

**Fig. 5:**
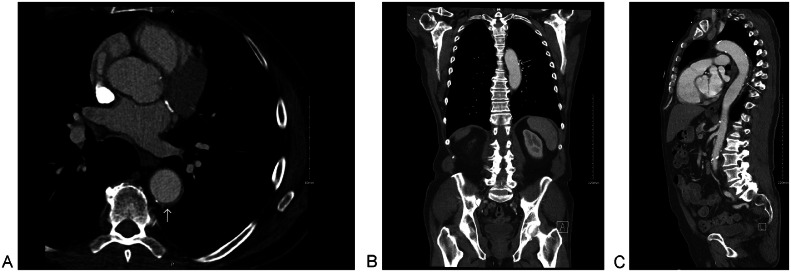
CT Angiogram of whole aorta after 4 months showing further reduction of TBIMH. (A) Axial at the level of the main pulmonary trunk. (B) Coronal at the level of the posterior mediastinum and (C) Sagittal para Medline.

The patient underwent serial CT angiography over 3 years. At the 3-month follow-up, CT angiogram of the whole aorta demonstrated a reduction in the size of the TBIMH from 33 mm to 13 mm, with a decrease in the maximum mural thrombus thickness from 14 mm to 9.5 mm (Fig. 4). A further reduction was also observed over 4 months (Fig. 5).

**Fig. 6:**
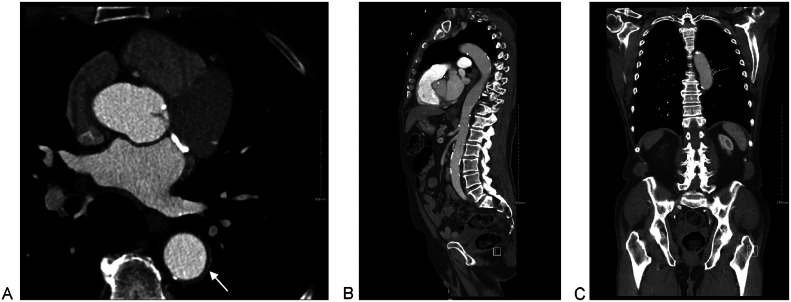
CT Angiogram of whole aorta after 3 years showing a significant resolution (white arrow). (A) Axial cut. (B) Sagittal cut and (C) Coronal cut.

At the 3-year follow-up, the patient remained medically stable, fully independent, and free of chest or back pain. Imaging showed significant resolution of the TBIMH (Fig. 6).

## Discussion

This case highlights the diagnostic journey of TBIMH in an elderly male who presented with mild, pleuritic posterior chest pain after exertion (gardening). Despite symptom improvement with analgesia and normal initial laboratory tests, the combination of marked hypertension and a broad cardiopulmonary differential prompted CT angiography, which confirmed the diagnosis. This report is educational for 3 reasons. First, it highlights that TBIMH may present atypically with mild pain, which can delay consideration of AAS in older patients. Second, it demonstrates the value of early and repeated CT angiography in confirming diagnosis, excluding dissection, and informing clinical decisions. Third, it provides extended imaging follow-up (up to 3 years) documenting progressive regression and near-resolution with medical management.

Most IMH patients present with sudden, severe anterior or posterior chest pain, often radiating to the back or neck [7,8]. In our case, the patient experienced mild pain in the back of the chest after gardening, emphasizing the importance of considering AAS in the elderly with atypical presentations. While most cases are symptomatic, IMH may also be detected incidentally [8]. When symptomatic, its clinical features may resemble those of classic dissection, although fewer than 5% of patients present with the classical signs and symptoms [7]. Compared to patients with dissection, those with IMH are typically older (mean age 69 vs 62 years), more frequently male, and more likely to have hypertension or atherosclerosis [9,10] similar to our patient. Pathologically, IMH is thought to result from spontaneous rupture of the vasa vasorum in the medial layer, promoted by wall stress, fragile vessels, chronic hypertension, and inflammation [3].

From a diagnostic perspective, imaging is central to confirming and differentiating it from other AAS [[11], [12], [13]]. A crescentic or circular aortic wall thickening without an intimal flap or tear is a hallmark for the diagnosis of IMH [12]. In practice, the radiological diagnostic work-up for stable patients often starts with a CXR, which may show a widened mediastinum. However, a normal CXR does not rule out AAS and may delay the diagnosis as it has a low diagnostic yield [13]. In unstable patients, echocardiography is the preferred modality, as it allows direct visualization of the intima, with Doppler useful for detecting flow communication; a characteristic finding is an echo-lucent area within a thickened aortic wall [11,12]. For stable patients, CT is the gold standard, with both unenhanced and contrast-enhanced phases required [14]. On noncontrast CT, IMH appears as a crescentic or circumferential hyperattenuating aortic wall thickening greater than 7 mm with attenuation of 60-70 HU, which does not enhance postcontrast [12,14]. In our patient, the initial thoracic aortogram demonstrated ectasia of the ascending aorta (44 mm) and a 41 mm descending thoracic aorta with a posterior intraluminal thrombus containing a hyperdense component consistent with TBIMH, without evidence of dissection.

In terms of follow-up, serial imaging is essential in medically managed TBIMH through CT or MRI, with MRI often favoured for longer-term surveillance to reduce cumulative radiation exposure [3,9]. The natural course of IMH varies: it may regress, remain stable, or progress to rupture or dissection. Unlike classic dissection, many IMH cases show complete resorption over time, with only a minority progressing to dissection [10,12]. Long-term observational data suggest a generally favourable prognosis with initial medical management; in Kaji et al. [15], in-hospital mortality was 0% in TBIMH. Nonetheless, clinically significant aortic events can occur. Moizumi et al. [16] reported a decline in event-free survival over time, with most aortic events occurring within 5 years. They also observed that early events were mainly driven by progression to overt dissection or enlargement of an ulcer-like projection, whereas later events were more often related to progressive aortic dilatation or rupture [16]. Kaji et al. [15] found that older age (≥70 years) and new ulcer-like projection on CT were strong predictors of progression. More recently, a large single-centre cohort of medically managed TBIMH (1995-2022) reported that isolated IMH frequently stabilized or resolved, but approximately one-fifth required later intervention, particularly in higher-risk presentations such as concomitant aneurysm or dissection [17]. Baseline CT predictors have also been proposed, Sueyoshi et al. [18] reported that maximum aortic diameter ≥40 mm and maximum aortic wall thickness ≥10 mm independently predicted progression in medically treated TBIMH.

In our case, continued conservative management was supported by serial CT angiography showing (i) no intimal flap/tear or new dissection, (ii) no features of rupture or impending rupture, (iii) stable aortic diameters on short-interval imaging, and (iv) reduction in TBIMH measurements. On Day 3 CTA, the intramural haematoma remained stable, with recanalisation and a focal contrast “pocket” connected to a left intercostal artery. Current CTA phenotyping distinguishes ulcer-like projections (visible communication with the aortic lumen) from intramural blood pools (enhancing foci connected to branch vessels without clear luminal communication) [19]; in this context, the intercostal-artery connection is more compatible with an intramural blood pool, supporting close surveillance rather than immediate intervention in a clinically stable patient [19]. Follow-up imaging then showed regression at 3 months (intramural haematoma thickness 13 mm; thrombus thickness 9.5 mm, from 31 mm and 14 mm initially), further improvement at 4 months, and near-resolution by 3 years, providing an objective “response-to-therapy” signal reinforcing ongoing conservative management.

This management aligns with the ESC recommendations. The 2024 ESC Guidelines emphasise immediate anti-impulse therapy in AAS, targeting systolic blood pressure <120 mmHg and heart rate ≤60 bpm, using intravenous beta-blockade first-line (with vasodilators added if required), alongside analgesia and close monitoring [20]. For TBIMH, the Guidelines recommend initial medical therapy under careful surveillance and escalation to endovascular repair when complicated features develop [20]. Complicated intramural hematoma includes recurrent pain, expansion, peri-aortic hematoma, and intimal disruption [20], none of which were present in our patient throughout the earlier and later surveillance. The Guidelines also recommend follow-up imaging after AAS using CT or MRI at 1, 3, 6, and 12 months and then annually if stable, with shorter intervals when concerning findings are present [20]. Our surveillance strategy followed this principle with early inpatient reassessment (Day 3), early outpatient imaging (3 and 4 months), and longer-term follow-up confirming durable regression at 3 years. Observational data further support risk stratification during the surveillance; Vaddavalli et al. [17] associated higher intervention rates with greater intramural hematoma thickness (around 7.2 mm) and larger aortic diameter (around 42 mm), and other series report a notable early medical-therapy failure rate associated with IMH thickness ≥8mm and IMH growth, highlighting the need for close monitoring and short-interval re-imaging in the acute phase [21].

For complicated TBIMH (rupture, impending rupture, or clinical deterioration), thoracic endovascular aortic repair is recommended [22]. In contemporary TEVAR series of complicated TBIMH, “complicated” disease is commonly defined by features such as persistent pain despite optimal blood pressure management, rapid dilatation, hemothorax or peri-aortic hemorrhage, ulcer-like projection, and other signs of impending rupture, with maximum aortic diameter ≥55 mm and/or short-interval IMH progression also used as intervention thresholds [23]. Tolboom et al. [23] reported TEVAR can be effective in preventing rupture in complicated TBIMH, but early risks (including stroke) and the need for later re-intervention underscore careful patient selection and structured postintervention surveillance.

## Conclusion

Type B aortic intramural hematoma can present with mild, atypical chest or back pain, and clinicians should maintain suspicion for acute aortic syndrome in older patients with marked hypertension despite reassuring initial tests. In this case, CT angiography was pivotal for diagnosis and, importantly, for excluding dissection and monitoring interval change. Serial imaging demonstrated stability on short-interval reassessment and progressive regression over time, supporting continued conservative management. The extended follow-up to 3 years in our patient provides additional reassurance that carefully selected, clinically stable patients can achieve durable resolution with medical therapy alone when supported by appropriate radiographic monitoring.

## Patient consent

We, the authors, certify that we have obtained written informed consent from the patient to publish our case report, including accompanying images. The patient has been encouraged to ask questions and has received satisfactory answers to all his inquiries. He had also been told to feel free to withdraw the study at any time before publication.
